# Development and Evaluation of a Clinical Prediction Model for Acute Kidney Injury Risk in Elderly Patients with Heart Failure with Reduced Ejection Fraction

**DOI:** 10.5334/gh.1577

**Published:** 2026-08-14

**Authors:** Rui Yang, Qiqi Song, Haohan Ma, Yongyang Fan, Shu Lin, Tao Liang, Jian Chen, Ning Tan, Lei Jiang

**Affiliations:** 1 School of Medicine, South China University of Technology, Guangzhou 510006, China; 2 Department of Cardiology, Guangdong Cardiovascular Institute, Guangdong Provincial People’s Hospital (Guangdong Academy of Medical Sciences), Southern Medical University, Guangzhou 510080, China; 3 Department of Cardiology, Third Affiliated Hospital of Guangzhou Medical University, Guangzhou 510150, China; 4 SDU-ANU Joint Science College, Shandong University, Weihai 264209, China; 5 Department of General Surgery, People’s Hospital of Yingde City, Yingde, Guangdong, China

**Keywords:** Heart Failure with Reduced Ejection Fraction, Acute Kidney Injury, Clinical prediction model

## Abstract

**Background:**

Acute kidney injury (AKI) is a significant complication among patients with heart failure with reduced ejection fraction (HFrEF). Once AKI occurs, therapeutic options are limited to supportive care, underscoring the clinical importance of early identification of patients at risk. This study sought to identify independent predictors of AKI development in geriatric HFrEF patients and subsequently establish a clinically applicable bedside risk quantification.

**Method:**

A total of 1,496 elderly patients (≥60 years) diagnosed with HFrEF at Guangdong Provincial People’s Hospital from January 2010 to December 2024 were enrolled according to predefined inclusion/exclusion criteria and stratified into an AKI group (n = 300) and a non-AKI group (n = 1196). Relevant parameters were screened using LASSO (Least Absolute Shrinkage and Selection Operator) regression, and risk factors for AKI in HFrEF patients were identified through univariate and multivariate logistic regression analyses. A nomogram was developed based on multivariate logistic regression results, accompanied by a corresponding heatmap. The predictive accuracy of the nomogram was evaluated using receiver operating characteristic (ROC) curves and calibration plots, while its clinical utility was demonstrated through decision curve analysis (DCA).

**Result:**

This study identified four independent predictors of AKI in patients with HFrEF, including N-terminal pro-B-type natriuretic peptide (NT-proBNP), uric acid (UA), CRP (C-reaction protein) and urea. A nomogram was developed based on these factors. The evaluation results demonstrated that the model exhibited good diagnostic accuracy, with an area under the receiver operating characteristic curve (AUC) of 0.721 (95% CI: 0.658–0.785). The calibration curve and DCA further indicated that the model possesses favorable clinical applicability.

**Conclusion:**

This study developed and validated a nomogram model for predicting AKI in patients with HFrEF. Clinicians can utilize this model to optimize clinical decision-making based on individualized patient characteristics, enabling personalized risk stratification for therapeutic interventions and prognostic evaluations.

## Introduction

Heart failure with reduced ejection fraction (HFrEF), conventionally defined as left ventricular ejection fraction (LVEF) ≤40%, represents a major clinical subtype of chronic heart failure driven by impaired systolic myocardial contractility. Characterized by inadequate ventricular pump function, HFrEF arises from diverse underlying etiologies, most prominently ischemic heart disease, dilated cardiomyopathy, hypertensive heart disease, and toxic myocardial injury. Patients with HFrEF often have multiple serious complications due to abnormal ventricular remodeling and hemodynamics, among which acute kidney injury (AKI) is one of the key factors leading to worse prognosis (1). Studies have shown that in HFrEF patients, decreased cardiac output, renal hypoperfusion, and neuroendocrine activations, such as overactivation of the renin-angiotensin system, can lead to decreased glomerular filtration rate (GFR) and ischemic renal tubule injury, thereby inducing AKI (2). AKI poses a markedly detrimental impact on patients with HFrEF (3). AKI can further aggravate the heart-kidney interaction injury, resulting in increased fluid retention, electrolyte disturbance, and metabolic acidosis, thus inducing malignant arrhythmia or sudden cardiac death (4). Consequently, AKI was significantly positively associated with all-cause mortality in HFrEF patients (5).

Although current guidelines emphasize the importance of monitoring renal function in patients with HFrEF, there is still no specific predictive system based on multimodal data tailored to this population (6). In this study, we evaluated multiple factors as predictors of HFrEF AKI risk in a retrospective study aimed at developing and validating a simple, reliable, and accurate risk assessment tool for patients with HFrEF to assess the risk of acute kidney injury.

## Materials and Methods

### Study design and data collection

This retrospective cohort study consecutively enrolled elderly patients (age ≥60 years) diagnosed with HFrEF at Guangdong Provincial People’s Hospital from January 2010 to December 2024. Baseline clinical characteristics were collected by reviewing the electronic medical records from the first day of admission. In accordance with the American Heart Association scientific statement, 1,695 patients met the diagnostic criteria for HFrEF. Exclusion criteria included:

- pre-existing chronic kidney disease stages 4–5,
- the patient received at least one known nephrotoxic medication (e.g., aminoglycosides, non-steroidal anti-inflammatory drugs, vancomycin, or contrast agents) within seven days prior to the onset of acute kidney injury,
- presence of urinary tract infections or renal artery stenosis,
- patients with a prior implantation of a left ventricular assist device (LVAD) were identified,
- concomitant septic shock, sepsis, or multiorgan failure,
- history of cardiac transplantation, and
- missing critical laboratory data. Ultimately, 1496 patients were included in the final analysis.

**Figure 1:**
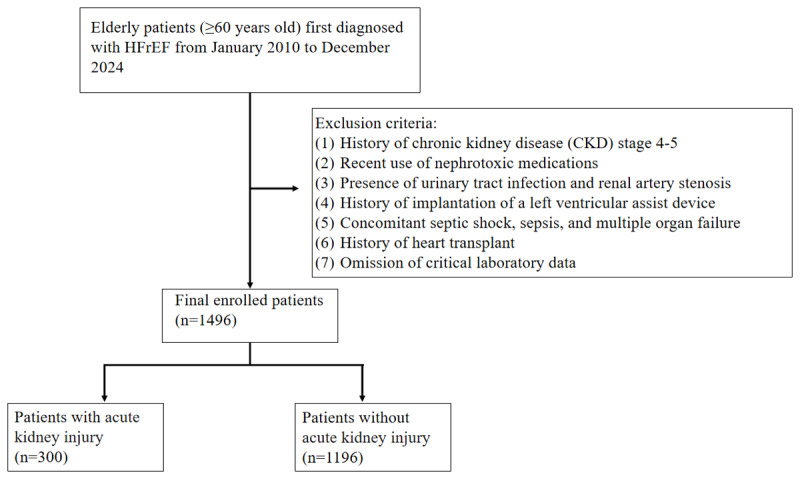
The flow chart of study population enrollment.

AKI was diagnosed based on serum creatinine elevation and oliguria criteria per KDIGO guidelines (7). The study enrollment flowchart is presented in Figure 1.

This study was approved by the Ethics Committee of Guangdong Provincial People’s Hospital with a waiver of written informed consent. Oral informed consent was obtained from conscious patients and all vulnerable patients’ guardian/next of kin by telephone and recorded by trained nurses during the follow-up period. However, all patient data were handled and maintained with strict confidentiality in compliance with the Declaration of Helsinki.

### Data source

Baseline characteristics, medical history, and laboratory results were collected from the electronic medical database. Clinical information was collected from an electronic case report form by one researcher and independently confirmed by another researcher.

### Statistical analysis

The study cohort was stratified into non-acute kidney injury (non-AKI) and AKI groups based on predefined inclusion/exclusion criteria. Comparative analysis of clinical characteristics between groups was performed using the ‘table1’ package in the R programming environment (version 4.2.1). Receiver operating characteristic (ROC) curve analysis was conducted via the ‘pROC’ package to calculate area under the curve (AUC) values, evaluating each variable’s discriminative capacity. Collinearity assessment was implemented through correlation heatmaps generated by the ‘corrplot’ package, calculating Spearman’s rank correlation coefficients (ρ) with a prespecified threshold of ρ < 0.7. Feature selection was performed using least absolute shrinkage and selection operator (LASSO) regression via the ‘glmnet’ package to identify optimal predictors. To facilitate clinical applicability, the selected predictors were weighted according to their regression coefficients, and a nomogram was constructed using the ‘rms’ package for visualization. The study cohort was then randomly divided into a training set and a testing set for model performance evaluation. Discrimination was quantified using ROC curve analysis, calibration was assessed via calibration slope, and clinical utility was evaluated using decision curve analysis (DCA) across a range of probability thresholds.

## Results

### Clinical characteristics

Table 1 presents the baseline characteristics of the study population, encompassing demographic profiles, clinical history, laboratory parameters, and echocardiographic data. Among the 1496 enrolled patients with HFrEF, the cohort was stratified into AKI (n = 300) and non-AKI (n = 1196) subgroups based on acute kidney injury comorbidity status. Notably, compared with the non-AKI group, patients in the AKI group exhibited a higher prevalence of a history of gout, as well as significantly elevated levels of urea, uric acid (UA), N-terminal pro-B-type natriuretic peptide (NT-proBNP), C-reactive protein (CRP), creatine kinase (CK), and high-sensitivity cardiac troponin (hs-cTn), while serum albumin concentrations were significantly lower (all P < 0.05).

**Table 1:** Baseline characteristics between non-AKI group and AKI group.


VARIABLE	NON-AKI (N = 1196)	AKI (N = 300)	TOTAL (N = 1496)	p-VALUE

**General information**				

Male, n (%)	774 (65)	216 (72)	990 (66)	0.021

Age, y	67 (63, 72)	68 (64, 74)	67 (63, 72)	0.05

**History**				

Smoking history, n (%)	310 (26)	84 (28)	394 (26)	0.51

Valvular Heart Disease, n (%)	521 (44)	124 (41)	645 (43)	0.528

Cerebral stroke, n (%)	76 (6)	30 (10)	106 (7)	0.038

NYHA Class IV heart failure, n (%)	154 (13)	58 (19)	212 (14)	0.006

Gout, n (%)	48 (4)	35 (12)	83 (6)	<0.001

Hypertension, n (%)	395 (33)	109 (36)	504 (34)	0.31

Diabetes, n (%)	247 (21)	85 (28)	332 (22)	0.005

Rheumatism, n (%)	10 (1)	5 (2)	15 (1)	0.199

**Parameters**				

Serum Inorganic phosphorus, mmol/L	1.18 (1.06, 1.32)	1.2 (1.07, 1.45)	1.18 (1.06, 1.34)	0.006

Urea, mmol/L	6.9 (5.4, 8.9)	9.93 (7.08, 15.03)	7.3 (5.6, 9.81)	<0.001

Uric acid, μmol/L	449.1 (361.39, 551.7)	534.05 (417.8, 662.92)	464.65 (373, 575.02)	<0.001

LDH, U/L	198 (169, 237)	230.9 (189.75, 291.25)	204 (171, 249)	<0.001

CK, U/L	78 (55, 115)	91 (60.83, 138.5)	80 (56, 120)	<0.001

CK-MB, U/L	10.2 (7.77, 13.9)	11.1 (8.4, 15.5)	10.25 (7.88, 14.1)	0.023

AST, U/L	26 (20.17, 34)	28 (22, 43)	26 (21, 35)	<0.001

TC, mmol/L	4.37 (3.67, 5.13)	3.99 (3.24, 4.69)	4.3 (3.59, 5.08)	<0.001

LDL-C, mmol/L	2.71 (2.2, 3.31)	2.6 (1.97, 3.12)	2.69 (2.16, 3.29)	0.001

ApoA-I, g/L	1.13 (0.94, 1.32)	1.04 (0.85, 1.19)	1.12 (0.93, 1.3)	<0.001

HDL-C, mmol/L	1.03 (0.85, 1.23)	0.94 (0.76, 1.13)	1.02 (0.84, 1.21)	<0.001

ADA, U/L	10.4 (8.5, 13.22)	11.7 (8.9, 14.72)	10.7 (8.5, 13.7)	0.005

GGT, U/L	43 (26, 76.88)	49 (31, 103)	44 (27, 81)	<0.001

TBIL, μmol/L	16.5 (12.2, 23.8)	18.7 (12.6, 29.15)	16.85 (12.3, 24.6)	0.002

TP, g/L	65.52 ± 6.94	64.79 ± 6.8	65.37 ± 6.91	0.101

TSH, mIU/L	1.59 (0.94, 2.54)	1.79 (1.06, 3.21)	1.63 (0.94, 2.67)	0.009

NT-proBNP, pg/mL	2363 (1005.75, 5343.25)	5712 (2335.75, 15659)	2917.5 (1172.5, 6308)	<0.001

hs-cTn, ng/L	20.4 (11.97, 36.28)	37.35 (19.07, 69.17)	22.3 (12.68, 42.7)	<0.001

Neutrophil count, 10^9^/L	5.1 (3.87, 6.58)	4.74 (3.63, 6.47)	4.99 (3.82, 6.53)	0.066

PDW, %	15.1 (12.2, 16.6)	15.85 (12.6, 16.72)	15.4 (12.3, 16.62)	0.045

Plateletcrit, %	1.9 (1.58, 2.4)	1.83 (1.43, 2.3)	1.9 (1.52, 2.4)	0.004

Platelet count,10^9^/L	195 (158, 238)	188 (146.45, 226)	194 (156, 236)	0.016

WBC count, 10^9^/L	6.85 (5.68, 8.23)	7.03 (5.56, 8.48)	6.87 (5.67, 8.29)	0.523

RDW, %	0.14 (0.13, 0.15)	0.14 (0.13, 0.16)	0.14 (0.13, 0.15)	0.028

RBC count, 10^12^/L	4.45 (4.05, 4.89)	4.38 (3.98, 4.84)	4.44 (4.02, 4.89)	0.07

Lymphocyte count, 10^9^/L	1.57 (1.19, 2.05)	1.52 (1.17, 2.06)	1.57 (1.19, 2.06)	0.606

Eosinophil count, 10^9^/L	0.12 (0.06, 0.21)	0.14 (0.06, 0.24)	0.12 (0.06, 0.22)	0.158

TT,s	16.4 (15.6, 17.3)	16.4 (15.7, 17.1)	16.4 (15.7, 17.2)	0.245

Fibrinogen, g/L	3.54 (2.97, 4.31)	3.48 (3.01, 4.03)	3.51 (2.98, 4.29)	0.242

PCT, ng/ml	0.05 (0.05, 0.06)	0.05 (0.05, 0.1)	0.05 (0.05, 0.07)	0.112

**Echocardiogram**				

Left Atrial Size, mm	43 (38, 48)	45 (40, 51)	43 (38.75, 48)	<0.001

RVVD, mm	58 (52, 63)	61 (54.75, 65)	59 (53, 64)	<0.001

LVEF, %	29 (24, 33)	29 (23.5, 34)	29 (24, 33.45)	0.606


### Univariate analysis

**Figure 2:**
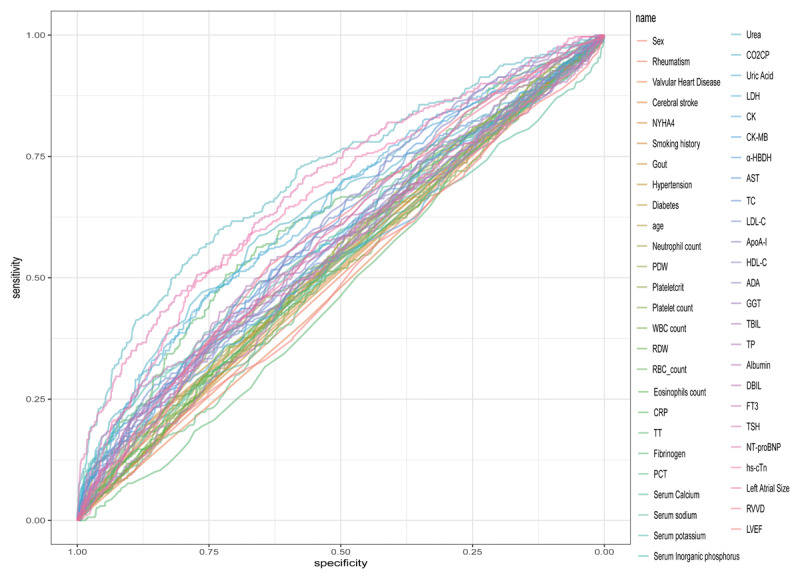
Univariate analysis showed the association between individual variables and the incidence of AKI.

Univariate analysis was conducted to evaluate demographic characteristics, medical history, laboratory parameters, and echocardiographic data (Figure 2). However, univariate findings suggested potential multifactorial interactions among covariates. Multivariable analysis was therefore implemented to systematically characterize variable interdependencies and their synergistic effects on outcome prediction, thereby enhancing model accuracy and reliability.

### Correlation heat map

**Figure 3:**
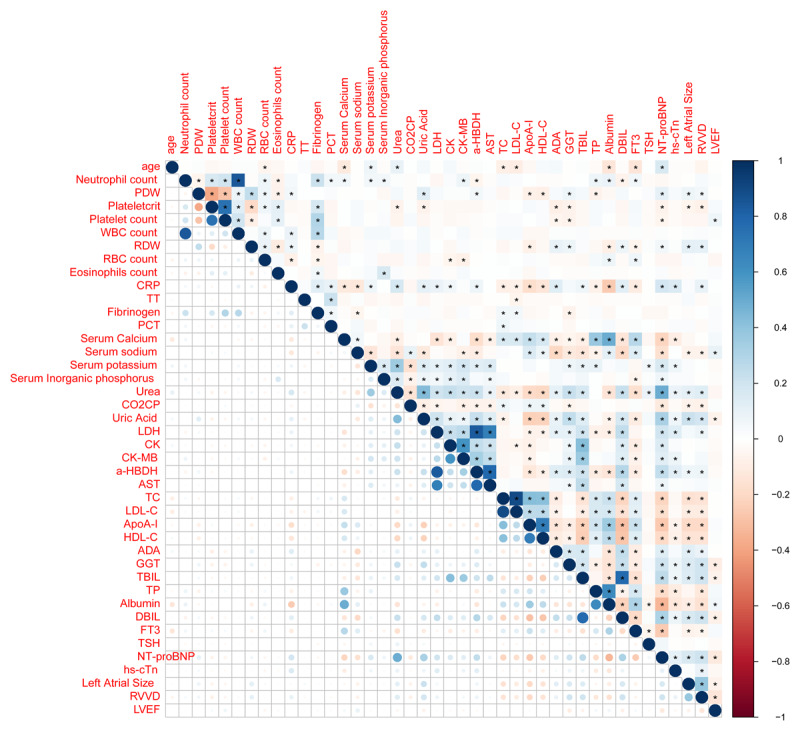
Collinearity analysis of the independent variables. The results highlight the presence of significant multicollinearity, suggesting the need for careful consideration when incorporating these variables into subsequent machine learning analyses to mitigate potential biases arising from collinearity. *P < 0.05.

The correlation heatmap (Figure 3) revealed varying degrees of intrinsic associations among covariates, underscoring the necessity for predictive model simplification through dimensionality reduction techniques. This optimization process aims to eliminate collinearity interference and enhance diagnostic precision in stratifying AKI susceptibility among HFrEF patients.

### Machine learning and variable selection

**Figure 4:**
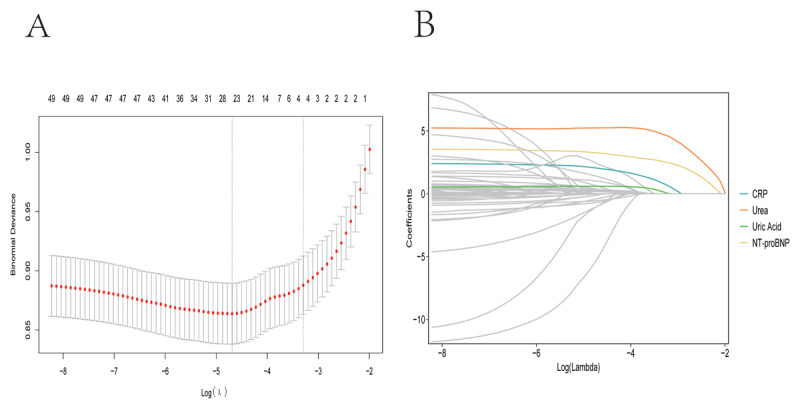
LASSO regression analysis for variable selection in predicting AKI in elderly patients with heart failure with reduced ejection fraction. Panel A shows the deviance (binomial) as a function of the log of the regularization parameter λ. The red dots represent the deviance at each λ value, with vertical dotted lines indicating the selected λ values based on cross-validation. Panel B shows the subset of variables with non-zero coefficients, identifying four potential risk factors associated with the outcome: NT-proBNP, Uric Acid (UA), C-reactive protein (CRP), and Urea. These variables were subsequently included in the multivariable analysis for further evaluation.

LASSO regression was implemented to screen 51 candidate variables, identifying factors associated with AKI in HFrEF patients (Figure 4A). Variable selection via LASSO regression was based on coefficient shrinkage to zero, indicating limited predictive relevance for excluded parameters. Variables retaining non-zero coefficients were designated as potential risk factors and advanced to multivariable logistic regression analysis (Figure 4B). Ultimately, four biomarkers—urea, UA, CRP, and NT-proBNP—emerged as significant predictors, demonstrating strong associations with AKI development in elderly HFrEF populations.

### Multivariable analysis and nomogram development

**Figure 5:**
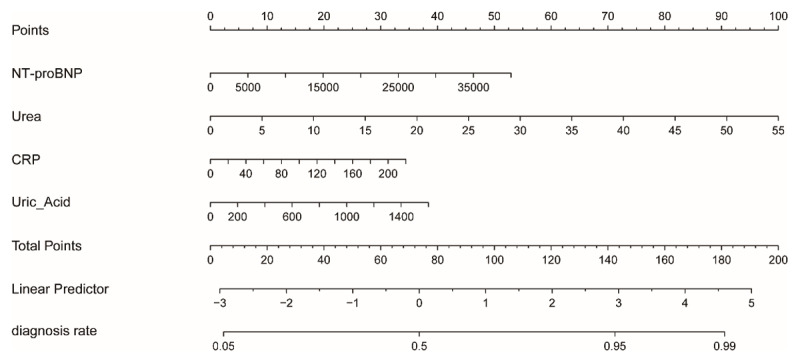
Nomogram representation for predicting the probability of the outcome. This visual tool incorporates the four selected variables, enabling individualized risk assessment to facilitate clinical decision-making. The nomogram offers a user-friendly interface, allowing healthcare professionals to gauge the likelihood of the outcome based on the specific values of the incorporated predictors.

Correlation heatmap analysis and multivariable logistic regression were performed to investigate associations between clinical variables and AKI development in HFrEF patients. Following confounder adjustment and model optimization, four independent predictors were identified: urea, UA, CRP, and NT-proBNP. A nomogram incorporating these predictors was developed as a visual predictive tool to estimate AKI probability (Figure 5). This model enables clinicians to perform individualized risk assessment for elderly patients with HFrEF based on a scoring system derived from four biomarkers. By quantifying the likelihood of AKI using these four predictive factors, the model provides a user-friendly tool to support clinical decision-making.

### Model evaluation

**Figure 6:**
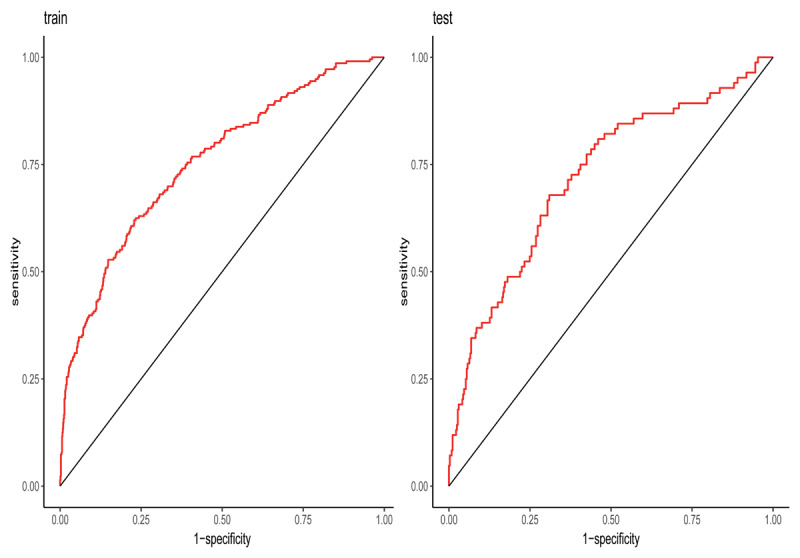
Receiver operating characteristic (ROC) curves evaluating the discriminative ability of the developed model. The ROC curve was used to evaluate the predictive performance of the model. The dataset was randomly divided into training and testing cohorts. In the training set, the area under the curve (AUC) was 0.754, indicating good discrimination. To assess the model’s generalizability to unseen data, it was further evaluated in the testing set, yielding an AUC of 0.721. These results demonstrate that the model has satisfactory predictive ability and generalization performance in both datasets.

**Figure 7:**
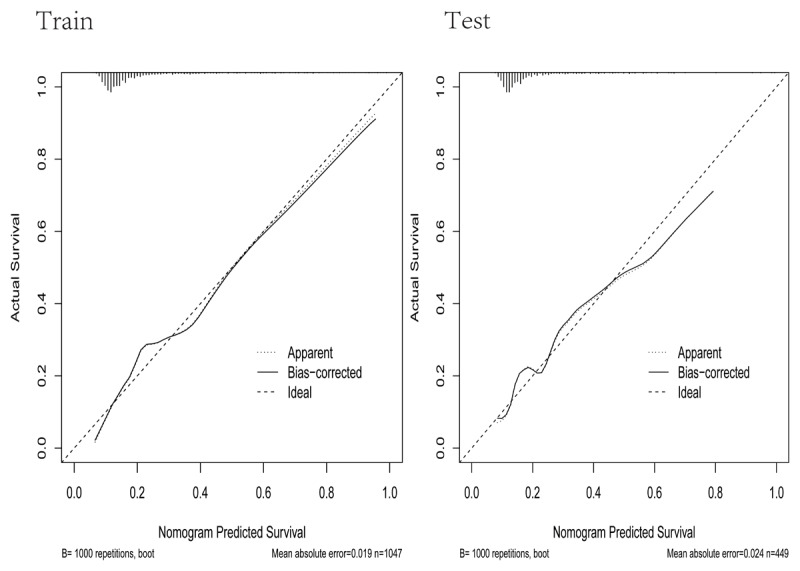
Calibration curve for evaluating the agreement between the model’s predicted probabilities and the observed outcomes. The calibration analysis resulted in a C-index of 0.754, signifying a robust concordance between predicted and actual outcomes. This indicates the model’s efficacy in accurately estimating the probabilities and ensuring reliable predictions for the outcome of interest.

**Figure 8:**
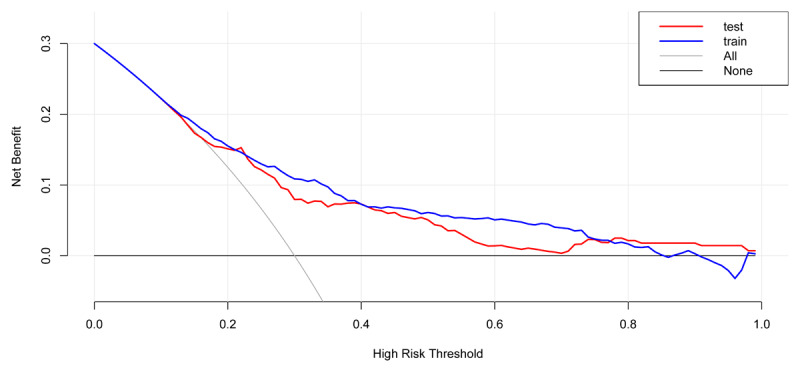
The DCA plots of the Nomogram for the risk of AKI in HFrEF patients.

Model validation was performed to evaluate the predictive performance of the developed nomogram. The study cohort was randomly divided into a training cohort (70% [1,047/1,496]) and a validation cohort (30% [449/14,96]). ROC curves were plotted to assess model discrimination, yielding an AUC of 0.754 (95% CI = 0.717–0.792) in the training set and 0.721 (95% CI = 0.658–0.785) in the validation set (Figure 6). Calibration curves were constructed for both cohorts (Figure 7), demonstrating good agreement between predicted probabilities and observed outcomes. These results confirm the nomogram’s accuracy in estimating AKI risk probability among HFrEF patients. The DCA (Figure 8) results demonstrated that the nomogram added more net benefit than the ‘treat all’ strategy or the ‘treat none’ strategy.

## Discussion

Based on the clinical data of elderly patients with HFrEF, this study developed and evaluated a clinical model for predicting the risk of concurrent AKI. Using LASSO regression for variable selection, the model was constructed in the form of a nomogram and systematically assessed through ROC analysis, calibration curves, and DCA (8). Four widely used clinical variables—urea, NT-proBNP, CRP, and UA—were ultimately included. By reanalyzing and integrating these conventional indicators, we proposed a cost-effective, easily implementable, and individualized risk assessment strategy with clear potential for clinical translation.

The selection of four biomarkers in this composite predictive model is pathophysiologically justified, as these indicators collectively underscore the critical role of cardiorenal crosstalk in mediating the development of AKI among patients with HFrEF (9). Mechanistically, these parameters reflect the intrinsic features of the AKI trajectory associated with HFrEF, including hemodynamic compromise, neurohormonal dysregulation, and disturbances in metabolic homeostasis (10).

From a biological and pathophysiological perspective, elevated urea levels often indicate impaired glomerular filtration (11), particularly under conditions of cardiogenic hypoperfusion (12). In such settings, urea elevation may precede changes in serum creatinine, offering early warning potential (13). In patients with HFrEF, reduced cardiac output (14) and abnormal distribution of circulating blood volume (15) can lead to renal hypoperfusion, making urea a key indicator for identifying the risk of prerenal AKI (16). NT-proBNP, an inactive cleavage product of the precursor of B-type natriuretic peptide (BNP), has a longer plasma half-life and is more strongly influenced by renal function compared to BNP (17). Thus, in the context of chronic heart failure or elderly populations, NT-proBNP is considered a more stable and reliable marker (18). In our study, elevated NT-proBNP levels were significantly associated with increased risk of AKI. This finding may be attributed to the reduced renal perfusion secondary to ventricular dilation and decreased cardiac output in HFrEF patients (19). NT-proBNP not only serves as an indicator of cardiac function but also reflects the imbalance of the cardiorenal axis, playing a crucial role in the early identification of AKI (20). Uric acid, traditionally regarded as a biomarker for hyperuricemia, gout, and metabolic syndrome (21), has recently been recognized as an independent risk factor for both chronic kidney disease (CKD) (22) and AKI (23). On one hand, elevated UA levels can induce apoptosis of renal tubular epithelial cells and oxidative stress (24); on the other hand, its accumulation may reflect early tubular dysfunction (25). The inclusion of UA in our predictive model highlights its clinical value in the early detection of AKI risk (26). Additionally, CRP, an acute-phase reactant (27), serves as a marker of systemic inflammation (28). Inflammatory processes contribute not only to the progression of HFrEF (29) but also to the pathogenesis of AKI via mechanisms such as microcirculatory dysfunction (30), endothelial injury (31), and tubular dysregulation (32). The incorporation of CRP into our model suggests that systemic inflammation may represent an independent risk factor for AKI in elderly patients with HFrEF (33).

Traditionally, these four biomarkers have been primarily used for disease monitoring or individual clinical judgment. In routine clinical practice, urea is commonly regarded as a key parameter for evaluating glomerular filtration (34), NT-proBNP as a central marker of heart failure severity (35), CRP as a classical indicator of systemic inflammation (36), and UA as a risk factor for metabolic disorders (37). However, in the specific context of elderly patients with HFrEF, the interplay between cardiac and renal function is particularly complex, and reliance on a single marker often fails to accurately identify individuals at high risk (38). To address this limitation, we employed LASSO regression algorithm to compress and refine a set of candidate variables, enabling the identification of the most predictive combination. Based on these results, we constructed a visualized nomogram that integrates these conventional biomarkers into a compact, high-performance, individualized risk scoring tool. This approach effectively repurposes widely available clinical indicators into a novel predictive model tailored to the unique pathophysiological characteristics of elderly HFrEF patients.

In the development of the clinical prediction model, LASSO regression was utilized to avoid the overfitting risk inherent in traditional multivariate regression, thereby ensuring a more refined and stable variable selection. The constructed nomogram presents an intuitive format that facilitates rapid bedside risk assessment by clinicians. The model’s performance was validated by ROC curves—with AUC values of 0.754 in the training set and 0.721 in the validation set—alongside calibration curves and DCA, demonstrating good discrimination, calibration, and clinical net benefit. These results indicate that the model possesses not only statistical significance but also practical clinical utility. Notably, the model relies solely on four routinely measured laboratory indicators without requiring costly imaging or novel biomarker assays, which enhances its applicability and cost-effectiveness. This makes it especially suitable for implementation in resource-limited primary care settings or geriatric wards, addressing the urgent need for precise risk prediction and early intervention among elderly patients with heart failure with reduced ejection fraction.

Previous studies have rarely focused on developing specific predictive models for AKI in elderly patients with HFrEF, with clinical practice often relying on subjective judgment or single biochemical markers to assess renal injury risk (39), which may lead to delayed diagnosis and intervention. The predictive model proposed in this study provides an objective and quantitative risk assessment tool tailored to this population, enabling clinicians to identify high-risk individuals prior to AKI onset and implement personalized management strategies such as medication adjustment, optimized fluid management, and dynamic monitoring of renal function. Compared to prior research, our study specifically targets the high-risk subgroup of elderly HFrEF patients, demonstrating strong population specificity. Methodologically, the study employed comprehensive validation and thorough performance evaluation. Furthermore, the included biomarkers are readily obtainable, facilitating rapid clinical application and dissemination. In contrast to models based on complex parameters, our model offers greater feasibility and generalizability.

While the clinical prediction model developed in this study demonstrates significant predictive capability, several inherent limitations warrant consideration. Firstly, the study employs a single-center, retrospective design with a long enrollment period (2010–2024). During this extended timeframe, significant evolution occurred in the management of heart failure, particularly regarding guideline-directed medical therapy. Although this extended period allows the study to reflect a broad spectrum of clinical practice, it also introduces heterogeneity in the therapeutic background of the enrolled patients, which may affect the model’s contemporaneity and generalizability.

Secondly, the prediction model is primarily constructed using baseline, routine laboratory indicators collected at hospital admission or initial consultation. It does not incorporate dynamic changes in these parameters during the treatment course. More critically, the model lacks detailed consideration of specific pharmacological treatments received by the patients, such as the use of diuretics, renin-angiotensin system inhibitors, angiotensin receptor-neprilysin inhibitors, and sodium-glucose cotransporter-2 inhibitors, and history of contrast agent exposure, which are all factors closely associated with the risk of acute kidney injury. Furthermore, the model has not explored the potential of emerging biomarkers, such as those from genomic studies (40). The absence of this information may constrain the model’s comprehensiveness and predictive accuracy.

Finally, the study participants were recruited from a specific medical center and focused on an elderly population with heart failure with reduced ejection fraction. Consequently, the model is most directly and reliably applicable to patient populations that closely resemble the derivation cohort in clinical characteristics. Before it can be applied to populations in different regions, healthcare settings, or with differing clinical features, rigorous external validation and calibration in appropriate prospective cohorts are essential to ensure its predictive performance and clinical applicability. Future research could further optimize the model’s performance by integrating longitudinal data, detailed treatment information, and multi-omics data.

## Conclusion

This study developed and validated a risk prediction model for AKI in elderly patients with HFrEF, based on four clinical indicators: urea, NT-proBNP, CRP, and UA. The model is simple in structure and stable in performance, demonstrating good visualization and clinical practicality. It holds potential for the early identification of high-risk patients within a similar clinical context and may assist in guiding individualized interventions. Further prospective, multicenter studies in comparable and broader populations are warranted to validate and optimize the model, thereby enhancing its generalizability and applicability.

## Data Availability

The datasets generated during and/or analyzed during the current study are not publicly available due to privacy or ethical restrictions but are available from the corresponding author on reasonable request.

## Abbreviations

HFrEF: heart failure with reduced ejection fraction; AKI: acute kidney injury; LDH: Lactate Dehydrogenase; CK: Creatine Kinase; CK-MB: Creatine Kinase-Muscle Brain; α-HBDH: Alpha-Hydroxybutyrate Dehydrogenase; AST: Aspartate Aminotransferase; TC: Total Cholesterol; LDL-C: Low-Density Lipoprotein Cholesterol; ApoA-I: Apolipoprotein A-I; HDL-C: High-Density Lipoprotein Cholesterol; ADA: Adenosine deaminase; GGT: Gamma-Glutamyl Transferase; TBIL: Total Bilirubin; TP: Total Protein; DBIL: Conjugated Bilirubin; FT3: Free Triiodothyronine; TSH: Thyroid-Stimulating Hormone; NT-proBNP: N-terminal pro-B-type natriuretic peptide; hs-cTn: High-Sensitivity Cardiac Troponin; PDW: Platelet Distribution Width; WBC: White Blood Cell; RDW: Red Blood Cell Distribution Width; RBC: Red Blood Cell; CRP: C-reactive protein; TT: Thrombin Time; PCT: Procalcitonin; CO_2_CP: Carbon Dioxide Combining Power; RVVD: Right Ventricular Vertical Diameter; LVEF: Left Ventricular Ejection Fraction.
